# Chloroplast Genome Sequence of Clusterbean (*Cyamopsis tetragonoloba* L.): Genome Structure and Comparative Analysis

**DOI:** 10.3390/genes8090212

**Published:** 2017-08-25

**Authors:** Tanvi Kaila, Pavan K. Chaduvla, Hukam C. Rawal, Swati Saxena, Anshika Tyagi, S. V. Amitha Mithra, Amolkumar U. Solanke, Pritam Kalia, T. R. Sharma, N. K. Singh, Kishor Gaikwad

**Affiliations:** 1 ICAR-National Research Centre on Plant Biotechnology, New Delhi 110012, India; tanvii88@gmail.com (T.K.); pavanraaz@gmail.com (P.K.C.); hukam.rawal@gmail.com (H.C.R.); swatisaxena605@gmail.com (S.S.); tyagi.anshika9@gmail.com (A.T.); amithamithra.nrcpb@gmail.com (S.V.A.M.); amolsgene@gmail.com (A.U.S.); trsharma1965@gmail.com (T.R.S.); nksingh@nrcpb.org (N.K.S.); 2 Division of Vegetable Sciences, Indian Agricultural Research Institute, Pusa, New Delhi 110012, India; pritam.kalia@gmail.com; 3 National Agri-Food Biotechnology Institute (NABI), Mohali, Punjab 140306, India

**Keywords:** clusterbean, Leguminosae, chloroplast genome, Illumina Hiseq 1000 platform, codon usage, microsatellites

## Abstract

Clusterbean (*Cyamopsis tetragonoloba* L.), also known as guar, belongs to the family Leguminosae, and is an annual herbaceous legume. Guar is the main source of galactomannan for gas mining industries. In the present study, the draft chloroplast genome of clusterbean was generated and compared to some of the previously reported legume chloroplast genomes. The chloroplast genome of clusterbean is 152,530 bp in length, with a quadripartite structure consisting of large single copy (LSC) and small single copy (SSC) of 83,025 bp and 17,879 bp in size, respectively, and a pair of inverted repeats (IRs) of 25,790 bp in size. The chloroplast genome contains 114 unique genes, which includes 78 protein coding genes, 30 tRNAs, 4 rRNAs genes, and 2 pseudogenes. It also harbors a 50 kb inversion, typical of the Leguminosae family. The IR region of the clusterbean chloroplast genome has undergone an expansion, and hence, the whole *rps19* gene is included in the IR, as compared to other legume plastid genomes. A total of 220 simple sequence repeats (SSRs) were detected in the clusterbean plastid genome. The analysis of the clusterbean plastid genome will provide useful insights for evolutionary, molecular and genetic engineering studies.

## 1. Introduction

Clusterbean (*Cyamopsis tetragonoloba* L.), also known as guar, is an annual herbaceous legume, tolerant to drought and salinity [1,2,3]. It belongs to the family Leguminosae and subfamily Papilionoideae. Due to its short growing season (90–120 days) [4], it is grown in rotation with other crops, like cotton, grain, sorghum, flax, etc. [5,6]. Guar also increases the nitrogen content and organic matter of the soil by the process of nitrogen fixation, and hence, leads to the increase in yield of other crops grown in rotation with it [6,7,8]. Guar is primarily cultivated in arid and semi-arid regions, like North-West India and South-East Pakistan. Guar pods are consumed as vegetables across the globe as they are a rich source of minerals, fibres, proteins and Vitamin C [9].

Guar is the main source of galactomannan for industries [10]. The endosperm of the guar seed is mainly composed of galactomannans. The galactomannan extracted from guar seed, known as guar gum [11], is used as a binding agent and stabiliser in industries like food, chemical, pharmaceuticals, cosmetic, etc. [8,12,13,14,15].

Chloroplasts are the organelles which provide energy to the plant by the process of photosynthesis [16]. Typically, the chloroplast (Cp) genome has a circular DNA, with a quadripartite structure having two copies of inverted repeats (IRs) separated by large single copy (LSC) and small single copy (SSC) region [17,18]. Generally, the size of the chloroplast genome varies between 120 kb to 160 kb in plants, and includes 110–130 genes, primarily involved in photosynthesis, transcription, and translation [19]. It has been proposed that the size of the Cp genome is influenced by the size of the IRs [20,21,22]. The genes present in the IR are replicated, and hence, present in duplicated copies [23]. There are several factors which can contribute to the size variation, but mainly the expansion/contraction or loss of IR has been reported as the evident factor. Another factor contributing to the variation in genome size is gene loss and gene duplication outside the IR [24]. However, loss of IRs has been reported in some legumes (IR-lacking clade, IRLC) [25,26] and coniferous Cp genomes [27]. Partial loss of IR also has been reported in black pine, which retains 495 bp of the IR [28]. The loss of IRs leads to a more dynamic arrangement of the Cp genome, thus undergoing gene losses and inversions in the single copy region, like in peas, as compared to the genome, which retains the IR and hence, is more stable [29,30]. Similarly, loss of intron has also been reported in genes like Clp protease (*clP*), ATP synthase (*atpF*), ribosomal proteins (*rps12*, *rpl2*, *rps16*) and RNA polymerase (*rpoC2*) [31].

Legume Cp genomes have undergone extensive rearrangements during their evolution, leading to a couple of inversions reported in the past, like 50 kb inversion and 78 kb inversion reported in subtribe Phaseolinae [32,33,34,35]. One feature by which the legume Cp genome is characterised is the 50 kb inversion in the LSC region, which is observed in most legumes like *Pisum sativum*, *Vigna radiata*, *Vicia faba* [29], *Glycine max* [36,37], *Cajanus cajan* [38], etc. Also, during the evolution of plants, many genes have been lost from the chloroplast. Amongst these losses, some were the transfer of Cp genes to the nucleus. Transfer of *rpl22* and *infA* gene to the nucleus has been reported in the legume genome, and hence, their nuclear copies are targeted to the chloroplast [39,40,41]. Similarly, transfer of *accD* gene to the nucleus has also been reported in the past [41]. Loss of intron from *rps12* and *clpP* has also been reported in the legume genome [35,39].

Even though chloroplast genomes have a conserved organisation, some variations are still observed in the plastid genome, like loss of *accD*, *psaI*, *rpl23*, *rps16*, *ycf4*, and *infA* genes. Also, duplication of some tRNA genes, *ycf2*, *rpl23*, and *psbA*, have been reported in the past [31,41]. Besides the loss of genes, various genes are also being reported as pseudogenes. Pseudogenes are genes that have stop codons in the protein coding sequence. The genes like *ycf2* [42,43], *infA*, *rpl23* [44], *rpl33*, *rps16*, *ycf15*, and *ycf68* [38] are the reported pseudogenes. Also, gene gain is a rare phenomenon, and not observed as frequently as gene loss in the plastid genome, as only three genes were gained in the plastome (*matK*, *ycf1*, *ycf2*), whereas many have been lost or transferred to the nucleus from the plastome [30].

Since the first reports of the complete sequencing of the Cp genome of tobacco [45] and liverwort [46], the interest in mining useful genomic information from Cp genome has increased, and as a result, 1139 Cp genome sequences of land plants are now available in NCBI Organelle Genome Resources database.

As the chloroplast genomes have a conserved gene content and organisation, and are maternally inherited [47], they serve as a valuable source for undertaking phylogenetic and evolutionary studies [48,49]. Also, due to very low levels of recombination and substitution rates, as compared to nuclear genomes, chloroplast genomes serve as useful genetic markers for phylogenetic analysis [50,51,52]. Chloroplast genomes are also used for DNA barcoding and breeding in agriculture [53]. A common strategy used for sequencing of the plastid genome is the use of a universal set of primers to amplify the whole Cp genome, followed by sequencing [54,55,56], or, the whole genome sequencing is done, in which total genomic DNA data is used to extract plastid genome sequences [57], like grapevine Cp genome sequence, which was obtained during the sequencing of the whole genome [58]. Next generation techniques have an advantage over labour intensive and low throughput cloning techniques, as enriched or un-enriched DNA can be used directly for sequencing [30]. The first attempt to use Next Generation Sequencing technology (454 GS 20 system) for the sequencing of Cp genome, was made by Moore et al. (2006) [59]. With the advent of Next Generation Sequencing, a large number of Cp genomes have now been sequenced [38,60,61,62]. Herbarium genomics has also been reported to be a promising field with respect to organelle genome sequencing [63]. Organelle genome sequencing is of great value to the branch of phylogenomics. Like genome skimming, which involves sequencing of chloroplast, mitochondrial, or rDNA, can be used to recover matrilineal genealogy [64]. Multiple platforms are available for sequencing of Cp genome, but Illumina is the most used platform for sequencing of chloroplast genomes [55,65,66,67]. In this study, we used purified chloroplast DNA as a template for sequencing by Illumina HiSeq 1000 platform (San Diego, CA, USA). This is the first report of the guar chloroplast genome, and hence, would help in phylogenetic analysis, DNA barcoding, and breeding in future.

## 2. Materials and Methods

### 2.1. Plant Material and Chloroplast DNA Isolation

Guar (variety RGC 936) was used in this study. Fresh leaves were harvested from the plant and kept in dark for 48 h prior to Cp DNA isolation. The Cp DNA isolation from the leaves was performed as per Kirti et al. (1993) [68].

### 2.2. Chloroplast Genome Sequencing, Assembly, and Annotation

The plastid libraries were prepared by Illumina Nextera DNA library preparation kit (San Diego, CA, USA). Initially, 50 ng of the plastid DNA was tagmented, cleaned, and amplified, and libraries were prepared as per manufacturer’s protocol, with an average size of 500 bp. The quality check (QC) of the libraries were validated by Bioanalyzer, using DNA High sensitivity chips (Agilent Technologies, California, USA), and thereafter, the samples were run on Illumina Hiseq 1000 platform.

FastQC v0.11.5 was used to assess the per base quality of the raw reads (50,642,415). A Phred score of 30 was set as the threshold for filtering reads. Average length of the reads was 96 bp. All 50,642,415 paired-end raw reads passed the quality filter threshold of 30 Phred score. With CLC genomics (workbench 9.5.1), (CLC Bio, Arhus, Denmark) 5,882,271 (11.62%) of these reads were mapped using the chloroplast reference genome of *Glycine max* (*G. max*), and assembled at 23 k-mer (auto). The thus obtained large contigs were reassembled again by guidance based de novo assembly with *G. max* Cp genome to obtain an assembly containing the largest contig, >150 kb size and N50 of 90,670 bp. A BLASTN search-based approach was used to order the contigs against the *G. max* Cp genome, with >80% matches and gaps filled by filtered reads at 90% similarity over 50% length.

The annotation of the chloroplast genome was performed by Dual Organellar Genome Annotator (DOGMA) [69] and hence coding sequences (cds), rRNAs, and tRNAs were identified by using plastid genetic code and BLAST homology searches. The tRNAs were verified by online tRNAscan-SE 1.21 search serve [70]. The exact gene and exon boundaries were verified, and the start and stop codons were manually corrected.

The entire chloroplast genome sequence of *Cyamopsis tetragonoloba*, along with gene annotations was submitted to GenBank (accession number: MF352008).

### 2.3. Genome Analysis

Full alignments of clusterbean chloroplast genome were performed using mVISTA program [71] in Shuffle-LAGAN mode. Selected legume Cp genomes were retrieved from NCBI: *Cajanus cajan* (KU729879), *G*. *max* (NC_7942), *P. vulgaris* (NC_9259), *Cicer arietinum* (NC_11163), *V. radiata* (NC_13843), and *Medicago truncatula* (NC_003119), which were used as references.

The comparison of gene order between the chloroplast genomes of clusterbean, *Arabidopsis thaliana* (NC_000932), *G. max* (NC_7942), *P. vulgaris* (NC_9259), *C. arietinum* (NC_11163), *V. radiata* (NC_13843), and *M. truncatula* (NC_003119) was performed with MAUVE [72]. Codon usage was calculated for all exons of protein-coding genes with CodonW 1.4.4. Base composition was calculated by DNA/RNA base composition calculator [73].

### 2.4. Simple Sequence Repeats Analysis

Chloroplast microsatellites (CpSSRs) were identified in high quality sequence of clusterbean by using MISA perl script [74]. The identified cpSSRs included mononucleotide repeats ≥8 bases, dinucleotides ≥10 bases (five repeats), and trinucleotides and tetranucleotides ≥12 bases (four and three repeats respectively), pentanucleotide ≥15 bases (3 repeats), and hexanucleotides ≥18 bases (3 repeats).

## 3. Results and Discussion

### 3.1. Genome Features of Clusterbean Chloroplast Genome

**Figure 1:**
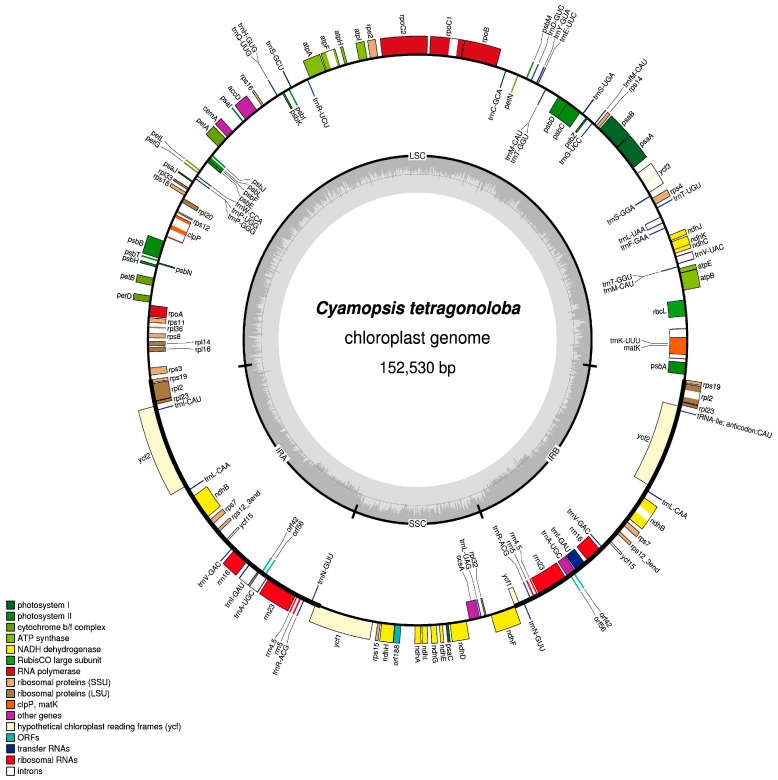
Map of *Cyamopsis tetragonoloba* plastid genome. Genes shown on the outside of the map are transcribed clockwise, while the genes that are shown on the inside are transcribed counterclockwise. The innermost darker gray corresponds to GC content, whereas the lighter gray corresponds to AT content. Different genes are colour coded. IR: inverted repeat; LSC: large single copy region; SSC: small single copy region.

The complete chloroplast genome of clusterbean is 152,530 bp in length. It has a typical quadripartite structure, with the Cp genome divided into LSC and SSC of 83,025 bp and 17,879 bp in size, respectively, and a pair of IRs of 25,790 bp in size (Figure 1). The size of the Cp genome is similar to other reported legume genomes (Supplementary Table S1). The GC content for the whole genome is 35%, which is in accordance with other reported legume genomes, like *Glycine max* [75], *Cicer arietinum* [33], *Vigna radiata* [76], and *Cajanus cajan* [38]. Similarly, the GC content for LSC, SSC, and IRs, is 33%, 29%, and 42%, respectively (Table 1). The high GC content for the IR regions can be attributed to the presence of four rRNAs genes (*rrn4.5*, *rrn5*, *rrn16*, *rrn23*), thus leading to sequence complexity and stabilisation of the whole genome.

The clusterbean chloroplast genome contains 114 unique genes when duplicated genes are counted only once, and includes 78 protein coding genes, 30 tRNAs, 4 rRNAs genes, and 2 pseudogenes. Individually, LSC contains 80 genes (57 protein coding genes, 22 tRNAs, and 1 pseudogene), SSC contains 13 genes (12 protein coding genes and 1 tRNA). The IR region consists of duplicated copies of 10 protein coding genes, 7 tRNAs, 4 rRNAs genes, and 1 pseudogene, therefore, in total, it consists of 22 genes (Table 2). The tRNA genes are distributed throughout the genome, and are encoded by 61 possible codons (excluding the stop codon). Duplicated tRNAs, trnM-CAU, and trnT-GGU, are present in the LSC region. Such tRNA duplications have been observed in the past in black pine, *Actinidia*, and pigeonpea [38,77,78]. In total, 12 intron-containing genes are present in the clusterbean Cp genome, out of which two (*ycf3* and *clpP*) contain two introns each (Supplementary Table S2). The *trnK*-UUU gene contains the largest intron (2573 bp), which also harbors the *matK* gene. Trans-splicing of *rps12* gene is observed in the clusterbean Cp genome, as is the case with other Cp genomes, like *Actinidia* [78] and *Pongamia pinnata* [79]. As a result of trans-splicing, 5′ exon is present in the LSC region, and the 3′ exon is duplicated in the IR region.

In clusterbean Cp genome, the protein coding region accounts for 52.7%, while the tRNA and rRNA coding regions account for 2.07% and 5.94%, respectively. The remaining genome consists of the intergenic region, introns, and pseudogenes.

Codon usage was calculated for the protein coding genes present in the clusterbean Cp genome. A total of 78 protein coding genes, comprising a length of 80,166 nucleotides, are represented by 26,722 codons (Table 3). As reported earlier also, leucine (2831 codons, 10.5% of the total) and cysteine (318 codons, 1.19% of the total) represent the most and least abundant amino acids, respectively [80,81,82]. Salim and Cavalcanti (2008) [83] suggested that there exists a relationship between codon usage bias and translational efficiency. They further explain that codon usage is biased towards either abundant tRNAs, or those codons which binds their cognate tRNAs more strongly than others. Also, the codon usage pattern in the clusterbean Cp genome is observed to be biased towards a high presentation of A or T at third codon position (Table 1), as supported by relative synonymous codon usage (RSCU) values. The value for codons ending with A and T is 40.42%, while codons ending with C and G is 12.61%. This bias towards the high presentation of A or T is also observed in other Cp genomes [82,84]. It has also been reported in the past that organellar proteins are encoded mainly by codons ending with A or U [85].

During the course of evolution, there have been many cases of intron and full gene losses among the angiosperms. Homologous recombination between intron-less cDNA and the original intron containing copy of DNA has been proposed as one of the mechanisms for the loss of introns. Loss of intron of *atpF* gene in Malpighiales was explained by the above mechanism [86,87]. Likewise, it has been reported that a clade known as IR-lacking clade (IRLC), which includes *Cicer arietinum*, *Medicago truncatula*, *Trifolium subterraneum*, *Pisum sativum*, and *Lathyrus sativus*, has lost *clpP* introns. Loss of intron from *rpl2* gene was also reported from various lineages of flowering plants [24]. Nevertheless, introns play an important role in gene expression, as the presence of an intron enhances the gene’s transcription. Also, introns present within the gene can be used as flanking sequences for the purpose of genetic engineering, thus providing efficient processing of foreign transcripts [53].

As is the case with intron loss, various gene losses have also been reported in angiosperms. Likewise, *rpl22* and *infA* genes are observed to be missing from clusterbean chloroplast genome. The independent transfer of *rpl22* gene to the nucleus has been reported in Fabaceae [40] and Fagaceae [88]. Similarly, transfer of *infA* gene to the nucleus has been documented in rosids, and was supported by the finding of expressed copies of the gene with stretches of chloroplast transit peptide in the nucleus [89]. The transfer of *rpl32* gene in Salicaceae [90,91] and *accD* gene in *Trifolium* [41] have also been well documented. The *accD* gene has been reported to be lost at least seven times in the course of evolution of angiosperms. In some plastid genomes, like *Medicago* and *Populus*, the phenomenon of nuclear substitution has been reported as a cause for loss of *rps16* gene from the plastome, as the nuclear encoded, mitochondrial copy of the gene is targeted to the plastid also [92]. But in the plastid genome of clusterbean, *rps16* gene has been found to be present as a pseudogene. Similarly, it is present as a pseudogene in Cp genome of pigeonpea [38], while its non-functional copy is present in *V.radiata* [76]. Loss of splicing activity might be the reason for it to be present as a pseudogene [93]. Also, *ycf15* is observed to be present as a pseudogene in clusterbean Cp genome. It has also been reported, in the past, to be present as a pseudogene in the Cp genome of pigeonpea [38], *Phaseolus vulgaris*, and *Vigna radiata* [33,76], as it contains premature stop codons within the coding sequence. It can be concluded from the above reports that increased rate of hypermutation has made the legumes more prone to rearrangements, and thus, more number of genes are lost or relocated to the nucleus in legumes [41,94].

### 3.2. Gene Order

**Figure 2:**
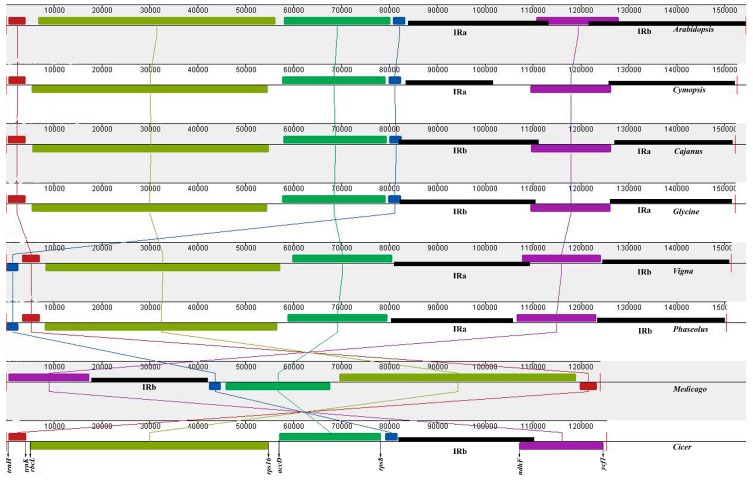
Gene order comparison of legume plastid genomes, with *Arabidopsis* Cp genome as reference, using MAUVE software. The boxes above the line represent the gene sequence in clockwise direction, and the boxes below the line represent gene sequences in the opposite orientation. The gene names at the bottom indicate the genes located at the boundaries of the boxes in Cp genome of pigeonpea.

Each of the sequenced legume Cp genomes possesses a unique structure. To deduce the structural homology, we compared the Cp genome of clusterbean with the sequenced legume genomes using MAUVE (Figure 2), taking *Arabidopsis* Cp genome as a reference. On comparison with *Arabidopsis*, it was found that the clusterbean and all the legume Cp genomes possesses a 50 kb inversion in LSC, spanning the region between the *rbcl* and *rps16* genes of the chloroplast.

The clusterbean Cp genome also possesses an additional inversion within the LSC and the IR region, occurring as a result of flip flop intramolecular recombination [95]. This inversion is also observed for *G. max* and pigeonpea Cp genome. The Cp genomes of *Cicer arietinum* and *Medicago truncatula* generally share the same gene order with clusterbean, except for the loss of IRb region in the former. This loss of IR region has been reported earlier too [96], and such legume tribes, lacking one IR region, form a new clade known as Inverted Repeat-lacking clade (IRLC) [97,98].

An inversion unique to subtribe phaseolinae, occurring as a result of expansion and subsequent contraction of IRs [99], is present in the Cp genome of *V. radiata* and *P. vulgaris*, but absent from other plastid genomes. This suggests that legume Cp genomes have undergone considerable rearrangements and diversification, and thus, provide a valuable resource for phylogenetic analysis.

### 3.3. Plastid Genome Sequence Comparison

**Figure 3:**
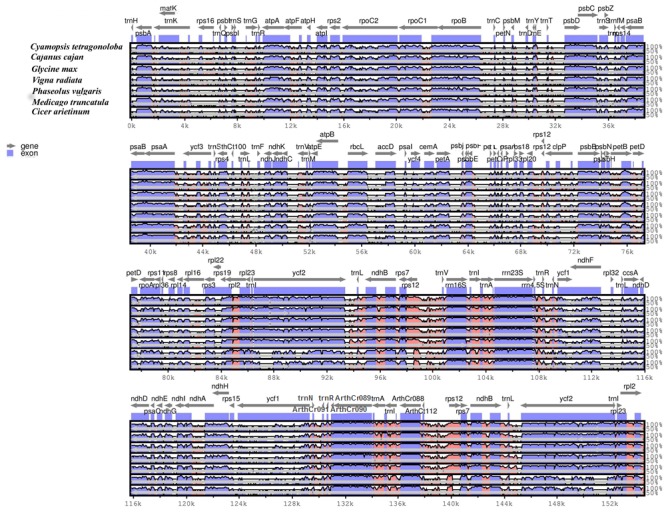
Sequence alignment of legume plastid genomes, with *C. tetragonoloba* Cp genome set as a reference using mVISTA. Position and transcriptional direction of each gene is indicated by gray arrows. Intergenic and genic regions are indicated by red and blue areas, respectively. Sequence identity between the Cp genomes is shown on y-axis as a percentage between 50% to 100%.

The availability of various legume plastid genomes provides the opportunity for comparison of Cp genomes. Hence, the sequence identity of the legume Cp genomes was plotted with the help of mVISTA (Figure 3), using annotations of clusterbean as reference. On alignment, it was found that the overall chloroplast genomes were conservative with some divergent regions. Similar to other plant species, coding regions were found to be more conservative than the non-coding regions. The most conserved region was the IR region, probably due to the presence of conserved rRNA genes and the phenomenon of copy correction [100]. The coding regions like *clpP*, *accD*, *petA*, *petD*, and *cemA* show high a degree of divergence, while the intergenic region between the genes *rpoB-psbD*, *ndhC-atpB*, *psbE-psbB*, *petD-rps3*, *trnK-UUU-rbcl*, and *ndhJ-ycf3*, also show a high degree of divergence.

### 3.4. Comparison of Inverted Repeat Boundaries of Clusterbean with Other Closely Related Plastid Genomes

**Figure 4:**
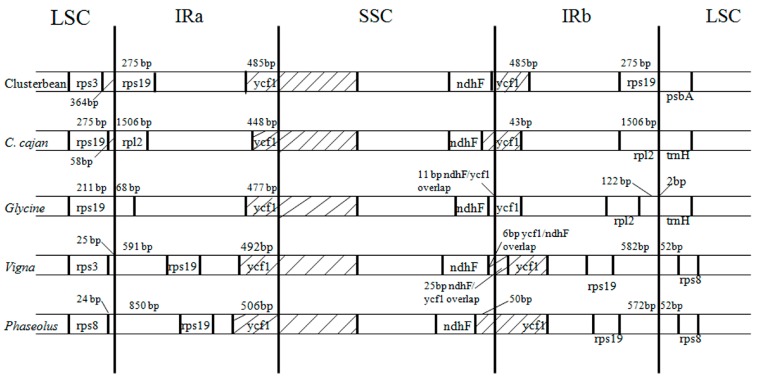
Comparison of the border positions of LSC, SSC and IR regions among the legume genomes. Genes are denoted by boxes, and the gaps between the genes and the boundaries are indicated by number of bases, unless the gene coincides with the boundary. Extensions of the genes are also indicated above the boxes.

The IR regions are known to promote the stability of the rest of the genome by intramolecular recombination between the two copies of inverted repeats, and thus, limiting the recombination between the two single copy regions [97,101]. The contraction and expansion of IRs leads to the size variation of the plastid genomes among the angiosperms. The comparison of the boundaries of clusterbean Cp genome with other plastid genomes is presented in Figure 4. The IR region of clusterbean contains 22 completely duplicated genes. At the IR/LSC junction, *rps19* gene is included in the IR region, and hence, is completely duplicated. On the other hand, at the IR/SSC junction, 485 bp of *ycf1* gene is included in the IR. As a result, a partial *ycf1* gene is included at the IRa/SSC junction, while the complete *ycf1* gene is included in the IR at the SSC/IRb junction. In comparison to other genomes, these boundaries fluctuate, like in *G.max* Cp genome, where 68 bp of *rps19* gene is included in the IR. Meanwhile, complete duplication of the *rps19* gene is seen in *Vigna radiata* and *Phaseolus vulgaris*, in contrast with the absence of *rps19* in the IR regions of pigeonpea. On the other hand, *ycf1* gene is included in the IRs of all the compared legumes, but the size varies among them. On comparison with other closely related legumes, the IR region of clusterbean (25,790 bp) was found to be smaller than that of *Vigna radiata* (26,474 bp), but larger than the IR region of *Cajanus cajan* (25,398 bp).

### 3.5. Simple Sequence Repeats Analysis

**Figure 5:**
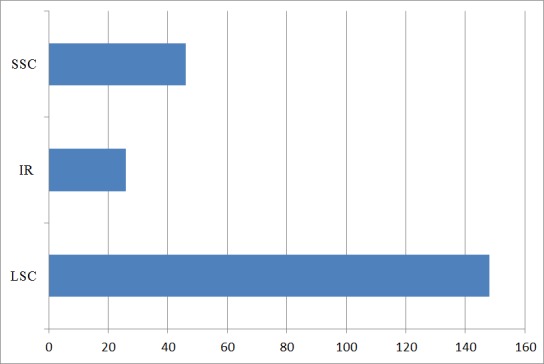
Simple sequence repeats (SSRs) distribution in three different regions: LSC, SSC and IR region. X-axis represents the number of SSRs.

**Figure 6:**
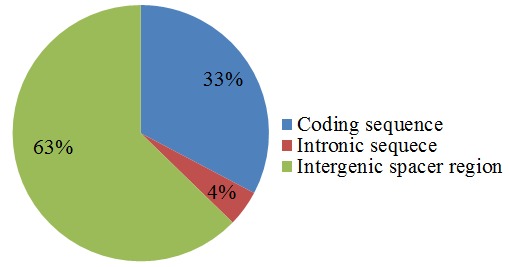
Repeat distribution among three different regions: coding sequences, intronic sequences, and intergenic spacer regions.

A group of tandem repeat sequences consisting of 1–6 nucleotide repeat units are known as simple sequence repeats (SSRs), or microsatellites [102]. CpSSRs are known to be relatively abundant, and demonstrate high reproducibility and polymorphism. Thus, these are frequently used in species identification and genetic analysis. Chloroplast SSRs were extracted using MISA perl script, and a total of 220 SSRs were detected in the clusterbean Cp genome (Supplementary Table S3). The numbers of SSR loci found are similar to that reported in *Vigna radiata*, but less than that reported in pigeonpea [88,103]. Among 220 SSRs reported, 67.27% (148 SSRs) are present in LSC region, 11.81% (26 SSRs) are present in IR region, and 20.9% (46 SSRs) are present in the SSC region (Figure 5). The findings were similar to that reported in artichoke [104] and *Datura stramonium* Cp genome [80]. Further, the SSRs were distributed among the coding, non-coding, and intergenic regions. Additionally, it was found that 33% (72) of the SSRs were present in the coding region, 4% (10 SSRs) in the intronic region and 63% (138) were present in the intergenic region (Figure 6). Also on comparison between coding and non-coding region, a higher number of SSRs were found to be present in the non-coding region than the coding region, making the results consistent with those observed for *G. max* [105] and *Datura stramonium* [80].

**Figure 7:**
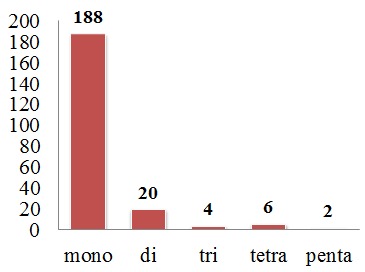
SSR distribution on the basis of repeat type. Y-axis represents the number of SSRs.

**Figure 8:**
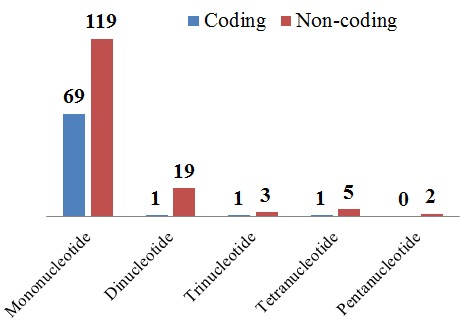
SSR type distribution between coding and non-coding regions. Y-axis represents the number of SSRs.

On the basis of the arrangement of nucleotides in the repeat motif, 78% of the SSRs were found to be perfect repeats, while 15%, 6%, and 1% were found to be compound interrupted, imperfect, and compound repeats, respectively. The microsatellites were further analysed on the basis of repeat types. The most abundant repeat type was mononucleotide, and the least abundant was pentanucleotide, with no hexanucleotide motifs detected (Figure 7). These repeat types were distributed among the coding and non-coding region (Figure 8). On analysis of these repeat types, it was found that mono, di, and trinucleotide repeats were mainly composed of A or T nucleotides. Wheeler et al. (2014) [106] also reported that the majority of mononucleotides are A/T rich. This bias in base composition is also consistent with overall AT richness in the clusterbean plastid genome. Such A/T rich repeats were also reported in *Camellia* species, *Sesame indicum*, *Glycine* species and *Sesamum indicum* [105,107,108,109].

Among the coding sequences, maximum numbers of repeats were detected in the *ycf1* gene region. In recent studies, *ycf1* gene is considered as the most variable locus [80,110]. The finding is consistent with others reported from *Glycine* species, *V. radiata*, *Camellia* species, *Cynaracardunculus* [76,104,105,108].

## 4. Conclusions

The clusterbean plastid genome was sequenced on an Illumina Hi-Seq 1000 platform, and the assembly was done using CLC Genomics Workbench 9.5.1. The genome is 152,530 bp in length, with a typical quadripartite structure, and consists of 114 unique genes, similar to other reported legume plastid genomes. This is the first study reporting the draft chloroplast genome sequence of clusterbean. The plastid genome of clusterbean, on comparison with other legumes, shows similar organisation, except for the IR expansion, where *rps19* is included in the IRs, and hence, completely duplicated. It also consists of two pseudogenes, namely *rps16* and *ycf15*. Gene loss is also observed, as the genes *rpl22* and *infA* are absent from the plastid genome. On doing SSR analysis, 220 SSR loci were found, with most SSRs present in the intergenic region. This study would be helpful in evolutionary and molecular studies.

## Figures and Tables

**Table 1:** Features of the chloroplast genome of *Cyamopsis tetragonoloba*. T: Thymine; U: Uridine; C; Cytosine; A: Adenine; G: Guanosine; IRa: Inverted Repeat a; IRb: Inverted Repeat b.

Features	T/U%	C%	A%	G%	Length (bp)	AT%
Genome	32	17	32	18	152,530	65
LSC	34	16	34	17	83,025	67
SSC	35	14	36	15	17,879	71
IRa/IRb	29	20	29	22	25,790	58
Prt.Coding genes	32	17	31	19	80,166	64
tRNA	26	23	22	29	3172	48
rRNA	19	23	26	31	9070	45
First position	24.1	18.4	31.5	25.8	26,722	55.6
Second position	33.2	19.9	29.6	17.1	26,722	62.8
Third position	39.1	12.9	30.0	14.7	26,722	69.1

**Table 2:** List of genes present in the Cp genome of clusterbean.

Category	Gene Name
Photosystem I	*psaA*,*B*,*C*,*I*,*J*,*Ycf3 ^a^*
Photosystem II	*psbA*,*B*,*C*,*D*,*E*,*F*,*H*,*I*,*J*,*K*,*L*,*M*,*N*,*T*,*Z/lhbA*
Cytochrome b6/f	*petA*,*B*,*D*,*G*,*L*,*N*
ATP Synthase	*atpA*,*B*,*E*,*F ^b^*,*H*,*I*
Rubisco	*rbcL*
NADH Oxidoreductase	*ndhA*,*B ^b^*^,*c*^,*C*,*D*,*E*,*F*,*G*,*H*,*I*,*J*,*K*
Large subunit ribosomal proteins	*rpl2 ^b^*^,*c*^,*14*,*16*,*20*,*23 ^c^*,*32*,*33*,*36*
Small subunit ribosomal proteins	*rps2*,*3*,*4*,*7 ^c^*,*8*,*11*,*12 ^c^*^,*d*^,*14*,*15*,*16 ^e^*,*18*,*19*
RNAP	*rpoA*, *rpoB*, *C1 ^b^*, *C2*,
Other Proteins	*accD*, *ccsA*, *matK*, *cemA*, *clpP ^a^*
Proteins of unknown Function	*ycf1 ^c^*^,^, *ycf2 ^b^*^,*c*^, *ycf15 ^c^*^,*e*^, *orf42 ^c^*, *orf56 ^c^*, *orf188*
Ribosomal RNAs	*rrn23 ^c^*,*16 ^c^*,*5 ^c^*,*4.5 ^c^*
Transfer RNAs	*trnH(GUG)*, *K(UUU) ^b^*, *M(CAU)*, *T(GGU)*, *V(UAC) ^b^*, *F(GAA)*, *L(UAA) ^b^*, *T(UGU)*, *S(GGA)*, *fM(CAU)*, *G(UCC)*, *S(UGA)*, *E(UUC)*, *Y(GUA)*, *D(GUC)*, *C(GCA)*, *R(UCU)*, *S(GCU)*, *Q(UUG)*, *W(CCA)*, *P(UGG)*, *P(GGG)*, *I(CAU) ^c^*, *L(CAA) ^c^*, *V(GAC) ^c^*, *I(GAU) ^b^*^,*c*^, *A(UGC) ^b^*^,*c*^, *R(ACG) ^c^*, *N(GUU) ^c^*, *L(UAG)*

*^a^* Gene containing two introns; *^b^* Gene containing a single intron; *^c^* Two gene copies in the IRs; *^d^* Gene divided into two independent transcription units; *^e^* Pseudogenes. RNAP: RNA Polymerase.

**Table 3:** Codon Usage for *Cyamopsis tetragonoloba*.

Amino Acid	Codon	Count	RSCU	tRNA
Ala	GCG	127	0.09	trnA-UGC
Ala	GCA	396	0.29	
Ala	GCT	629	0.47	
Ala	GCC	193	0.14	
Cys	TGT	231	0.73	trnC-GCA
Cys	TGC	87	0.27	
Asp	GAT	836	0.80	trnD-GUC
Asp	GAC	211	0.20	
Glu	GAG	322	0.23	trnE-UUC
Glu	GAA	1051	0.77	
Phe	TTT	1106	0.68	trnF-GAA
Phe	TTC	509	0.32	
Gly	GGG	284	0.16	trnG-UCC
Gly	GGA	698	0.40	
Gly	GGT	588	0.34	
Gly	GGC	162	0.09	
His	CAT	511	0.79	trnH-GUG
His	CAC	136	0.21	
Ile	ATA	838	0.35	trnI-GAU
Ile	ATT	1188	0.49	trnI-CAU
Ile	ATC	401	0.17	
Lys	AAG	335	0.22	trnK-UUU
Lys	AAA	1185	0.78	
Leu	TTG	561	0.20	trnL-UAA
Leu	TTA	944	0.33	trnL-CAA
Leu	CTG	168	0.06	trnL-UAG
Leu	CTA	389	0.14	
Leu	CTT	590	0.21	
Leu	CTC	179	0.06	
Met	ATG	607	1.00	trnM-CAU
Asn	AAT	1051	0.78	trnN-GUU
Asn	AAC	291	0.22	
Pro	CCG	128	0.12	trnP-GGG
Pro	CCA	339	0.31	trnP-UGG
Pro	CCT	406	0.37	
Pro	CCC	211	0.19	
Gln	CAG	204	0.21	trnQ-UUG
Gln	CAA	764	0.79	
Arg	AGG	159	0.10	trnR-UCU
Arg	AGA	494	0.32	trnR-ACG
Arg	CGG	105	0.07	
Arg	CGA	364	0.23	
Arg	CGT	347	0.22	
Arg	CGC	91	0.06	
Ser	AGT	404	0.20	trnS-UGA
Ser	AGC	121	0.06	trnS-GGA
Ser	TCG	181	0.09	trnS-GCU
Ser	TCA	442	0.21	
Ser	TCT	604	0.29	
Ser	TCC	306	0.15	
Thr	ACG	136	0.10	trnT-UGU
Thr	ACA	424	0.31	trnT-GGU
Thr	ACT	577	0.43	
Thr	ACC	219	0.16	
Val	GTG	175	0.12	trnV-UAC
Val	GTA	540	0.38	trnV-GAC
Val	GTT	540	0.38	
Val	GTC	162	0.11	
Trp	TGG	448	1.00	trnW-CCA
Tyr	TAT	852	0.83	trnY-GUA
Tyr	TAC	170	0.17	
Ter	TGA	3	0.60	
Ter	TAG	2	0.40	
Ter	TAA	0	0.00	

RSCU: relative synonymous codon usage.

## Supplementary Materials

The following are available online at www.mdpi.com/2073-4425/8/9/212/s1. Table S1: Size comparison among six Cp genomes completely sequenced in legumes; Table S2: List of genes with introns in Cp genome of *Cyamopsis tetragonoloba*; Table S3: SSRs found in *Cyamopsis tetragonoloba*.

## Abbreviations

Cp	chloroplast
LSC	large single-copy region
SSC	small single-copy region
IR	inverted repeat
DNA	deoxyribonucleic acid
bp	base pair(s)
GC	guanine–cytosine
AT	adenosine–thymine
kb	kilobase(s)
RNA	ribonucleic acid
rRNA	ribosomal RNA
SSR	simple sequence repeat(s)
tRNA	transfer RNA
