# Supplementary material for: Chloroplast Genome Sequence of Clusterbean (Cyamopsis tetragonoloba L.): Genome Structure and Comparative Analysis
Source: Genes (Basel). 2017 Aug 25;8(9):212. doi: 10.3390/genes8090212 (PMC5615346; doi:10.3390/genes8090212)
Supplement: Supplementary file 1 [file genes-08-00212-s001.zip › Supplementary Material/Supplementary Table 1.doc]

**Supplementary Table S1- Size comparison among six cp genomes completely sequenced in legumes**

| **Species** | **Accession Number** | **Genome size (bp)** | **LSC (bp)** | **SSC (bp)** | **IR (bp)** | **No. of unique genes** | **No. of inverted repeats** |
| --- | --- | --- | --- | --- | --- | --- | --- |
| *Glycine max* | NC_007942 | 152,218 | 83,175 | 17,895 | 25,574 | 111 | 2 |
| *Phaseolus vulgaris* | NC_009259 | 150,285 | 79,824 | 17,610 | 26,426 | 108 | 2 |
| *Vigna radiata* | NC_013843 | 151, 271 | 80,896 | 17,427 | 26,474 | 108 | 2 |
| *Cicer arietinum* | NC_011163 | 125,319 | - | - | 50,000 | 108 | 1 |
| *Cajanus cajan* | KU729879 | 152,242 | 83,455 | 17,871 | 25,398 | 116 | 2 |
| *Cyamopsis tetragonoloba* | MF352008 | 152,530 | 83,025 | 17,879 | 25,790 | 114 | 2 |
