# Supplementary material for: Chloroplast Genome Sequence of Clusterbean (Cyamopsis tetragonoloba L.): Genome Structure and Comparative Analysis
Source: Genes (Basel). 2017 Aug 25;8(9):212. doi: 10.3390/genes8090212 (PMC5615346; doi:10.3390/genes8090212)
Supplement: Supplementary file 1 [file genes-08-00212-s001.zip › Supplementary Material/Supplementary Table 2.doc]

**Supplementary Table S2- List of genes with introns in cp genome of *Cyamopsis tetragonoloba***

| S. No | Gene | Strand | Region | Start | End | Exon I (bp) | Intron I (bp) | Exon II (bp) | Intron II (bp) | Exon III (bp) |
| --- | --- | --- | --- | --- | --- | --- | --- | --- | --- | --- |
| 1 | *trnK-UUU* | - | LSC | 1696 | 4344 | 28 | 2584 | 36 | - | - |
| 2 | *trnV-UAC* | + | LSC | 9879 | 10540 | 38 | 587 | 36 | - | - |
| 3 | *trnL-UAA* | - | LSC | 13607 | 14201 | 49 | 509 | 36 | - | - |
| 4 | *ycf3* | + | LSC | 16488 | 18431 | 125 | 697 | 227 | 745 | 149 |
| 5 | *rpoC1* | + | LSC | 37547 | 40409 | 440 | 785 | 1637 | - | - |
| 6 | *atpF* | + | LSC | 48563 | 49853 | 167 | 713 | 410 | - | - |
| 7 | *clpP* | - | LSC | 68578 | 70651 | 224 | 775 | 296 | 710 | 68 |
| 8 | *rpl2* | - | IR a | 83359 | 84899 | 392 | 681 | 467 | - | - |
| 9 | *ycf2* | + | IR a | 85303 | 92665 | 305 | 197 | 6860 | - | - |
| 10 | *ndhB* | - | IR a | 93753 | 95973 | 752 | 692 | 776 | - | - |
| 11 | *trnI-GAU* | + | IR a | 101452 | 102476 | 41 | 949 | 34 | - | - |
| 12 | *trnA-UGC* | + | IR a | 102541 | 103423 | 37 | 811 | 34 | - | - |
| 13 | *trnA-UGC* | - | IR b | 132087 | 132969 | 37 | 811 | 34 | - | - |
| 14 | *trnI-GAU* | - | IR b | 133034 | 134058 | 41 | 949 | 34 | - | - |
| 15 | *ndhB* | + | IR b | 139537 | 141757 | 752 | 692 | 776 | - | - |
| 16 | *ycf2* | - | IR b | 142845 | 150207 | 305 | 197 | 6860 | - | - |
| 17 | *rpl2* | + | IR b | 150611 | 152151 | 392 | 681 | 467 | - | - |
