# Supplementary material for: Chloroplast Genome Sequence of Clusterbean (Cyamopsis tetragonoloba L.): Genome Structure and Comparative Analysis
Source: Genes (Basel). 2017 Aug 25;8(9):212. doi: 10.3390/genes8090212 (PMC5615346; doi:10.3390/genes8090212)
Supplement: Supplementary file 1 [file genes-08-00212-s001.zip › Supplementary Material/Supplementary Table 3.doc]

**Supplementary Table S3- SSRs found in  *Cyamopsis tetragonoloba***

| **SSR No.** | **SSR motif** | **SSR type** | **Size** | **Start** | **End** | ***Location*** | **Region** |
| --- | --- | --- | --- | --- | --- | --- | --- |
| 1 | (C)8(T)9 | c | 17 | 21 | 37 | *rps19-psbA* | spacer |
| 2 | (A)10 | p1 | 10 | 1961 | 1970 | *trnK(UUU)-matK* | spacer |
| 3 | (TCT)4 | p3 | 12 | 2181 | 2192 | *matK* | cds |
| 4 | (T)9 | p1 | 9 | 2322 | 2330 | *matK* | cds |
| 5 | (A)8N(A)8N(A)10 | i | 195 | 2864 | 3058 | *matk* | cds |
| 6 | (T)8 | p1 | 8 | 3796 | 3803 | *matk-trnK(UUU)* | spacer |
| 7 | (A)8 | p1 | 8 | 4015 | 4022 | *matk-trnK(UUU)* | spacer |
| 8 | (A)10ttct(A)9 | i | 23 | 4510 | 4532 | *trnK(UUU)-rbcl* | spacer |
| 9 | (AT)8aa(AT)9 | i | 36 | 5217 | 5252 | *trnK(UUU)-rbcl* | spacer |
| 10 | (T)12 | p1 | 12 | 7026 | 7037 | *rbcl-atpB* | spacer |
| 11 | (A)9 | p1 | 9 | 7253 | 7261 | *rbcl-atpB* | spacer |
| 12 | (A)10 | p1 | 10 | 7626 | 7635 | *atpB* | cds |
| 13 | (C)10(A)13 | c | 23 | 9702 | 9724 | *trnT(GGU)-trnV(UAC)* | spacer |
| 14 | (A)8 | p1 | 8 | 10435 | 10442 | *trnV(UAC) intron* | intron |
| 15 | (T)9 | p1 | 9 | 11880 | 11888 | *ndhK* | cds |
| 16 | (A)8 | p1 | 8 | 12805 | 12812 | *ndhJ-trnF(GAA)* | spacer |
| 17 | (T)8 | p1 | 8 | 12965 | 12972 | *ndhJ-trnF(GAA)* | spacer |
| 18 | (A)9 | p1 | 9 | 13274 | 13282 | *trnF(GAA)-trnL(UAA)* | spacer |
| 19 | (A)8 | p1 | 8 | 13404 | 13411 | *trnF(GAA)-trnL(UAA)* | spacer |
| 20 | (T)11 | p1 | 11 | 14370 | 14380 | *trnL(UAA)-trnT(UGU)* | spacer |
| 21 | (ATT)4 | p3 | 12 | 14671 | 14682 | *trnL(UAA)-trnT(UGU)* | spacer |
| 22 | (T)9 | p1 | 9 | 15161 | 15169 | *trnt(UGU)-rps4* | spacer |
| 23 | (A)8 | p1 | 8 | 16333 | 16340 | *trnS(GGA)-ycf3* | spacer |
| 24 | (T)8 | p1 | 8 | 17072 | 17079 | *ycf3 intron1* | intron |
| 25 | (TATC)3N(ATAG)3 | ci | 39 | 18480 | 18518 | *ycf3-psaA* | spacer |
| 26 | (T)13N(TA)8 | ci | 67 | 24661 | 24727 | *trnG(UCC)-psbZ* | spacer |
| 27 | (T)8 | p1 | 8 | 25215 | 25222 | *psbZ-trnS(UGA)* | spacer |
| 28 | (T)10 | p1 | 10 | 25593 | 25602 | *trns(GGA)-psbC* | spacer |
| 29 | (ATT)5N(A)8 | ci | 35 | 28688 | 28722 | *psbD-trnT(GGU)* | spacer |
| 30 | (T)8 | p1 | 8 | 29030 | 29037 | *psbD-trnT(GGU)* | spacer |
| 31 | (T)10 | p1 | 10 | 29253 | 29262 | *psbD-trnT(GGU)* | spacer |
| 32 | (AAT)4 | p3 | 12 | 29686 | 29697 | *trnM(CAU)-trnE(UUC)* | spacer |
| 33 | (T)8 | p1 | 8 | 30146 | 30153 | *trnM(CAU)-trnE(UUC)* | spacer |
| 34 | (T)9 | p1 | 9 | 30567 | 30575 | *trnY(GUA)-trnD(GUC)* | spacer |
| 35 | (T)8 | p1 | 8 | 30690 | 30697 | *trnY(GUA)-trnD(GUC)* | spacer |
| 36 | (A)8N(A)8 | i | 108 | 31002 | 31109 | *trnD(GUC)-psbM* | spacer |
| 37 | (A)8N(A)10N(T)9 | ci | 141 | 31506 | 31646 | *psbM-petN* | spacer |
| 38 | (TA)5 | p2 | 10 | 31797 | 31806 | *psbM-petN* | spacer |
| 39 | (T)8 | p1 | 8 | 32469 | 32476 | *petN-trnC(GCA)* | spacer |
| 40 | (A)8 | p1 | 8 | 32638 | 32645 | *petN-trnC(GCA)* | spacer |
| 41 | (T)8N(TA)5N(TA)7 | ci | 136 | 32770 | 32905 | *petN-trnC(GCA)* | spacer |
| 42 | (T)8ctattg(A)14 | ci | 28 | 33470 | 33497 | *trnC(GCA)-rpoB* | spacer |
| 43 | (A)9 | p1 | 9 | 33632 | 33640 | *trnC(GCA)-rpoB* | spacer |
| 44 | (AT)5 | p2 | 10 | 33746 | 33755 | *trnC(GCA)-rpoB* | spacer |
| 45 | (A)10N(A)8 | i | 114 | 34944 | 35057 | *rpoB* | cds |
| 46 | (A)8 | p1 | 8 | 37618 | 37625 | *rpoC1 exon1* | cds |
| 47 | (A)10 | p1 | 10 | 38211 | 38220 | *rpoC1 intron* | intron |
| 48 | (A)10 | p1 | 10 | 38561 | 38570 | *rpoC1 intron* | intron |
| 49 | (T)8 | p1 | 8 | 38682 | 38689 | *rpoC1 intron* | intron |
| 50 | (T)8 | p1 | 8 | 38878 | 38885 | *rpoC1 exon2* | cds |
| 51 | (A)9 | p1 | 9 | 40092 | 40100 | *rpoC1 exon2* | cds |
| 52 | (A)9 | p1 | 9 | 42175 | 42183 | *rpoC2* | cds |
| 53 | (T)8 | p1 | 8 | 42622 | 42629 | *rpoC2* | cds |
| 54 | (A)11N(A)9N(A)9N(A)8 | i | 179 | 42763 | 42941 | *rpoC2* | cds |
| 55 | (T)9 | p1 | 9 | 44734 | 44742 | *rpo2-rps2* | spacer |
| 56 | (A)10 | p1 | 10 | 45285 | 45294 | *rps2* | cds |
| 57 | (A)10 | p1 | 10 | 46953 | 46962 | *atpI-atpH* | spacer |
| 58 | (A)9 | p1 | 9 | 47664 | 47672 | *atpI-atpH* | spacer |
| 59 | (T)8 | p1 | 8 | 48328 | 48335 | *atpH-atpF* | spacer |
| 60 | (AT)6N(TA)5 | ci | 35 | 48466 | 48500 | *atpH-atpF* | spacer |
| 61 | (T)8 | p1 | 8 | 48687 | 48694 | *atpF exon1* | cds |
| 62 | (T)9 | p1 | 9 | 49032 | 49040 | *atpF intron* | intron |
| 63 | (A)8 | p1 | 8 | 49190 | 49197 | *atpF intron* | intron |
| 64 | (A)18 | p1 | 18 | 51582 | 51599 | *atpA-trnR(UCU)* | spacer |
| 65 | (T)13 | p1 | 13 | 51752 | 51764 | *trnR(UCU)-trnS(GCU)* | spacer |
| 66 | (A)8 | p1 | 8 | 51943 | 51950 | *trnR(UCU)-trnS(GCU)* | spacer |
| 67 | (T)8 | p1 | 8 | 52310 | 52317 | *trnR(UCU)-trnS(GCU)* | spacer |
| 68 | (T)9 | p1 | 9 | 52979 | 52987 | *trnR(UCU)-trnS(GCU)* | spacer |
| 69 | (A)8N(T)9 | ci | 50 | 53152 | 53201 | *trnR(UCU)-trnS(GCU)* | spacer |
| 70 | (T)9 | p1 | 9 | 53411 | 53419 | *trnS(GCU)-psbI* | spacer |
| 71 | (T)9 | p1 | 9 | 54050 | 54058 | *psbI-psbK* | spacer |
| 72 | (T)10N(TA)6N(A)9 | ci | 108 | 54310 | 54417 | *psbK-trnQ(UUG)* | spacer |
| 73 | (T)11 | p1 | 11 | 55016 | 55026 | *trnH(GUG)-rps16* | spacer |
| 74 | (T)8 | p1 | 8 | 56005 | 56012 | *trnH(GUG)-rps16* | spacer |
| 75 | (G)9 | p1 | 9 | 56163 | 56171 | *trnH(GUG)-rps16* | spacer |
| 76 | (A)8 | p1 | 8 | 56436 | 56443 | *rps16-accD* | spacer |
| 77 | (A)8N(T)10 | ci | 80 | 56663 | 56742 | *rps16-accD* | spacer |
| 78 | (A)9N(T)8 | ci | 51 | 57099 | 57149 | *accD* | cds |
| 79 | (A)12 | p1 | 12 | 58416 | 58427 | *accD-psaI* | spacer |
| 80 | (A)11cg(T)11 | ci | 24 | 59369 | 59392 | *psaI-cemA* | spacer |
| 81 | (A)8 | p1 | 8 | 59648 | 59655 | *psaI-cemA* | spacer |
| 82 | (A)9 | p1 | 9 | 61090 | 61098 | *petA* | cds |
| 83 | (A)8 | p1 | 8 | 61303 | 61310 | *petA* | cds |
| 84 | (A)12 | p1 | 12 | 62313 | 62324 | *petA-psbJ* | spacer |
| 85 | (A)10 | p1 | 10 | 62472 | 62481 | *petA-psbJ* | spacer |
| 86 | (AT)5N(T)8N(A)8 | ci | 99 | 65134 | 65232 | *trnP(UGG)-trnP(GGG)* | spacer |
| 87 | (T)8 | p1 | 8 | 65454 | 65461 | *psaJ* | cds |
| 88 | (T)8 | p1 | 8 | 65579 | 65586 | *psaJ-rpl13* | spacer |
| 89 | (A)10 | p1 | 10 | 66769 | 66778 | *rps18* | cds |
| 90 | (T)9 | p1 | 9 | 66918 | 66926 | *rps18-rpl20* | spacer |
| 91 | (A)10N(TA)5(T)9 | CI | 71 | 67503 | 67573 | *rpl20-rps12* | spacer |
| 92 | (T)8N(CTTT)3 | CI | 53 | 67694 | 67746 | *rpl20-rps12* | spacer |
| 93 | (TA)5 | p2 | 10 | 68125 | 68134 | *rpl20-rps12* | spacer |
| 94 | (AT)5 | p2 | 10 | 68373 | 68382 | *rpl12-clpP* | spacer |
| 95 | (T)9 | p1 | 9 | 69080 | 69088 | *clpP intron1* | intron |
| 96 | (T)9N(A)10 | CI | 75 | 69264 | 69338 | *clpP intron1* | intron |
| 97 | (T)11 | p1 | 11 | 70749 | 70759 | *clpP-psbB* | spacer |
| 98 | (G)8 | p1 | 8 | 71661 | 71668 | *psbB* | cds |
| 99 | (T)13 | p1 | 13 | 75712 | 75724 | *petB-petD* | spacer |
| 100 | (A)9N(T)9N(A)15 | ci | 175 | 76430 | 76604 | *petD-rpoA* | spacer |
| 101 | (ATCA)3N(AT)5N(T)8 | ci | 113 | 78343 | 78455 | *rps11-rpl36* | spacer |
| 102 | (T)8 | p1 | 8 | 78744 | 78751 | *rpl36-rps8* | spacer |
| 103 | (T)11 | p1 | 11 | 79007 | 79017 | *rpl36-rps8* | spacer |
| 104 | (T)11acgttctat(TA)5 | ci | 30 | 79540 | 79569 | *rps8-rpl14* | spacer |
| 105 | (TA)5 | p2 | 10 | 80766 | 80775 | *rpl16-rps3* | spacer |
| 106 | (A)11 | p1 | 11 | 81086 | 81096 | *rpl16-rps3* | spacer |
| 107 | (T)11 | p1 | 11 | 81354 | 81364 | *rpl16-rps3* | spacer |
| 108 | (T)8 | p1 | 8 | 81553 | 81560 | *rpl16-rps3* | spacer |
| 109 | (T)9 | p1 | 9 | 81679 | 81687 | *rpl16-rps3* | spacer |
| 110 | (A)9 | p1 | 9 | 81945 | 81953 | *rpl16-rps3* | spacer |
| 111 | (T)8 | p1 | 8 | 82357 | 82364 | *rps3* | cds |
| 112 | (T)12 | p1 | 12 | 82712 | 82723 | *rps3-rps19* | spacer |
| 113 | (A)9N(T)14 | CI | 102 | 82826 | 82927 | *rps3-rps19* | spacer |
| 114 | (T)9 | p1 | 9 | 83279 | 83287 | *rps19* | cds |
| 115 | (A)8 | p1 | 8 | 89019 | 89026 | *ycf2 exon2* | cds |
| 116 | (A)8 | p1 | 8 | 91386 | 91393 | *ycf2 exon2* | cds |
| 117 | (G)8 | p1 | 8 | 91973 | 91980 | *ycf2 exon2* | cds |
| 118 | (A)16 | p1 | 16 | 92679 | 92694 | *ycf2-trnL(CAA)* | spacer |
| 119 | (A)10 | p1 | 10 | 93349 | 93358 | *trnL(CAA)-ndhB* | spacer |
| 120 | (T)8 | p1 | 8 | 98004 | 98011 | *rps12-ycf15* | spacer |
| 121 | (A)8 | p1 | 8 | 103143 | 103150 | *orf56* | cds |
| 122 | (A)8N(T)9 | ci | 77 | 107409 | 107485 | *trnR(ACG)-trnN(GUU)* | spacer |
| 123 | (CAGAA)3 | p5 | 15 | 107759 | 107773 | *trnR(ACG)-trnN(GUU)* | spacer |
| 124 | (T)8 | p1 | 8 | 108753 | 108760 | *ycf1* | cds |
| 125 | (C)8N(A)8 | ci | 73 | 109041 | 109113 | *ycf1* | cds |
| 126 | (T)9 | p1 | 9 | 109368 | 109376 | *ycf1* | cds |
| 127 | (A)10N(A)8N(T)11 | ci | 148 | 109750 | 109897 | *ycf1* | cds |
| 128 | (A)8 | p1 | 8 | 110002 | 110009 | *ycf1* | cds |
| 129 | (A)13 | p1 | 13 | 110296 | 110308 | *ycf1* | cds |
| 130 | (A)10 | p1 | 10 | 110599 | 110608 | *ycf1* | cds |
| 131 | (A)8 | p1 | 8 | 110982 | 110989 | *ycf1* | cds |
| 132 | (A)11 | p1 | 11 | 111098 | 111108 | *ycf1* | cds |
| 133 | (A)13N(A)8N(A)11 | i | 153 | 111429 | 111581 | *ycf1* | cds |
| 134 | (T)11 | p1 | 11 | 111979 | 111989 | *ycf1* | cds |
| 135 | (A)11N(A)9 | i | 120 | 112277 | 112396 | *ycf1* | cds |
| 136 | (T)9N(A)8 | ci | 70 | 112660 | 112729 | *ycf1* | cds |
| 137 | (A)8 | p1 | 8 | 113353 | 113360 | *ycf1* | cds |
| 138 | (A)9 | p1 | 9 | 113556 | 113564 | *ycf1* | cds |
| 139 | (T)8N(T)8 | i | 88 | 113889 | 113976 | *ycf1-rps15* | spacer |
| 140 | (A)8 | p1 | 8 | 114181 | 114188 | *rps15* | cds |
| 141 | (T)10 | p1 | 10 | 114471 | 114480 | *rps15-ndhH* | spacer |
| 142 | (T)9 | p1 | 9 | 115735 | 115743 | *orf188* | cds |
| 143 | (T)8 | p1 | 8 | 116363 | 116370 | *orf188-ndhA* | spacer |
| 144 | (AT)5 | p2 | 10 | 116684 | 116693 | *orf188-ndhA* | spacer |
| 145 | (A)9 | p1 | 9 | 117007 | 117015 | *orf188-ndhA* | spacer |
| 146 | (TTTA)3 | p4 | 12 | 117242 | 117253 | *orf188-ndhA* | spacer |
| 147 | (A)8 | p1 | 8 | 118058 | 118065 | *ndhA-ndhI* | spacer |
| 148 | (AT)6 | p2 | 12 | 118707 | 118718 | *ndhI-ndhG* | spacer |
| 149 | (T)15N(T)8 | i | 89 | 119503 | 119591 | *ndhG-ndhE* | spacer |
| 150 | (ATCA)3 | p4 | 12 | 119975 | 119986 | *ndhE* | cds |
| 151 | (A)8 | p1 | 8 | 121577 | 121584 | *ndhD* | cds |
| 152 | (A)11 | p1 | 11 | 122198 | 122208 | *ndhD-ccsA* | spacer |
| 153 | (TA)5 | p2 | 10 | 122383 | 122392 | *ccsA* | cds |
| 154 | (A)8 | p1 | 8 | 123264 | 123271 | *ccsA* | cds |
| 155 | (T)8 | p1 | 8 | 124002 | 124009 | *rpl32* | cds |
| 156 | (A)10 | p1 | 10 | 124272 | 124281 | *rpll32-ndhF* | spacer |
| 157 | (T)8 | p1 | 8 | 125117 | 125124 | *ndhF* | cds |
| 158 | (A)9 | p1 | 9 | 125850 | 125858 | *ndhF* | cds |
| 159 | (T)8 | p1 | 8 | 126032 | 126039 | *ndhF* | cds |
| 160 | (T)8 | p1 | 8 | 126327 | 126334 | *ndhF* | cds |
| 161 | (A)8 | p1 | 8 | 126750 | 126757 | *ycf1* | cds |
| 162 | (TTCTG)3 | p5 | 15 | 127737 | 127751 | *trnN(GUU)-trnR(ACG)* | spacer |
| 163 | (A)9N(T)8 | ci | 77 | 128025 | 128101 | *trnN(GUU)-trnR(ACG)* | spacer |
| 164 | (T)8 | p1 | 8 | 132360 | 132367 | *orf56* | cds |
| 165 | (A)8 | p1 | 8 | 137499 | 137506 | *ycf5-rps12* | spacer |
| 166 | (T)10 | p1 | 10 | 142152 | 142161 | *ndhB-trnL(CAA)* | spacer |
| 167 | (T)16 | p1 | 16 | 142816 | 142831 | *trnL(CAA)-ycf2* | spacer |
| 168 | (C)8 | p1 | 8 | 143530 | 143537 | *ycf2 exon2* | cds |
| 169 | (T)8 | p1 | 8 | 144117 | 144124 | *ycf2 exon2* | cds |
| 170 | (T)8 | p1 | 8 | 146484 | 146491 | *ycf2 exon2* | cds |
| 171 | (A)9 | p1 | 9 | 152223 | 152231 | *rps19* | cds |

p- perfect repeat, i- imperfect repeat, c- compound repeat, ci- coumpound imperfect repeat.
